# Effectiveness of a psychological online training to promote physical activity among students: protocol of a randomized-controlled trial

**DOI:** 10.1186/s13063-021-05333-2

**Published:** 2021-06-21

**Authors:** Lena Violetta Krämer, Nadine Eschrig, Lena Keinhorst, Luisa Schöchlin, Lisa Stephan, Malin Stiene, Jürgen Bengel

**Affiliations:** Department of Rehabilitation Psychology and Psychotherapy, Institute of Psychology, University of Freiburg, Engelbergerstr. 41, 79085 Freiburg, Germany

**Keywords:** Physical activity, Exercise, students, E-health, Health Action Process Approach, RCT

## Abstract

**Background:**

Many students in Germany do not meet recommended amounts of physical activity. In order to promote physical activity in students, web-based interventions are increasingly implemented. Yet, data on effectiveness of web-based interventions in university students is low. Our study aims at investigating a web-based intervention for students. The intervention is based on the Health Action Process Approach (HAPA), which discriminates between processes of intention formation (motivational processes) and processes of intention implementation (volitional processes). Primary outcome is change in physical activity; secondary outcomes are motivational and volitional variables as proposed by the HAPA as well as quality of life and depressive symptoms.

**Methods:**

A two-armed randomized controlled trial (RCT) of parallel design is conducted. Participants are recruited via the internet platform StudiCare (www.studicare.com). After the baseline assessment (t1), participants are randomized to either intervention group (immediate access to web-based intervention) or control group (access only after follow-up assessment). Four weeks later, post-assessment (t2) is performed in both groups followed by a follow-up assessment (t3) 3 months later. Assessments take place online. Main outcome analyses will follow an intention-to-treat principle by including all randomized participants into the analyses. Outcomes will be analysed using a linear mixed model, assuming data are missing at random. The mixed model will include group, time, and the interaction of group and time as fixed effects and participant and university as random effect.

**Discussion:**

This study is a high-quality RCT with three assessment points and intention-to-treat analysis meeting the state-of-the-art of effectiveness studies. Recruitment covers almost 20 universities in three countries, leading to high external validity. The results of this study will be of great relevance for student health campaigns, as they reflect the effectiveness of self-help interventions for young adults with regard to behaviour change as well as motivational and volitional determinants. From a lifespan perspective, it is important to help students find their way into regular physical activity.

**Trial registration:**

The German clinical trials register (DRKS) DRKS00016889. Registered on 28 February 2019

## Background

Studying is a time of new tasks and stressors [1]. It is a time of performance pressure and lack of time as well as financial and future concerns [2]. Physical activity could help students reducing their stress reactivity [3], and it may serve as a potential health promotion resource for students [4]. Physical activity is a protective factor for the development of chronic medical diseases such as diabetes, cardiovascular disease, and cancer [5, 6]. And it can play an important role in the prevention and treatment of psychiatric diseases such as depression [7, 8]. Several meta-analyses have suggested that the effectiveness of regular exercise in reducing depressive symptoms is comparable to psychotherapeutic and pharmacotherapy treatments [7, 9].

However, many students in Germany do not meet the recommended amounts of physical activity [(cf. [10]): only 27.6% of students are physically active for at least 2.5 h with respect to physical activities making them sweat or breathe harder than normal [11]. Compared to a national study by Germany’s public health institute RKI (Robert Koch Institute) [12], students seem to be even less physically active than young adults in general. Within the RKI sample of young adults, 50% indicated to be physically active for at least 2.5 h a week.

In order to promote physical activity in students, web-based interventions are increasingly being implemented [13]. Web-based interventions are cost-effective [14] and allow for a flexible use in public health settings without spatial, personnel and time constraints [15]. Due to their high internet affinity [16], students are ideally suited as a target group for web-based trainings. The effectiveness of web-based health interventions has been proven for a variety of health and risk behaviours [15]. Meta-analyses show that web-based trainings can increase the level and frequency of physical activity with small to medium effect sizes [13, 17]. Although the effectiveness of web-based physical activity training in the general population is well documented, the data on the effectiveness in university students is low.

Most of the studies investigating the effectiveness of web-based trainings to promote physical activity in students show methodological shortcomings [13]. The most common limitations are the lack of randomization, intention-to-treat analyses, or follow-up measurements [(e.g. [18–21]). One exception is the randomized-controlled trial by Sriramatr and colleagues [22]: medium to large effect sizes of the web-based intervention were found for the change in leisure activities (d = 0.73) and high effect sizes for the daily number of steps (d = 1.41). The effects remained until the follow-up assessment after three months (d = 0.72; d = 1.25). However, the sample of this study only includes female students in Thailand. As the authors point out, culture characteristics may be a restriction for the external validity of the results. Another study investigates college females in the USA: Wadsworth and Hallam [21] find an intervention effect of d = 0.23 with regard to the frequency of moderate activities. Yet, no other measure of physical activity is reported (e.g. duration of physical activity, high intensity activities). Both studies [21, 22] investigate an intervention based on Social Cognitive Theory (SCT [23]). Indeed, most interventions in this field of research are based on SCT [17]. Another important theory of health behaviour change—the Health Action Process Approach (HAPA [24])—has not yet been investigated in web-based physical activity trials in students.

The HAPA goes beyond SCT by emphasizing post-intentional processes. It is based on the assumption that the process of changing a health behaviour includes a pre-intentional (motivational) and a post-intentional (volitional) phase. According to the model, an intention to be physically active is developed if more *positive consequences* (e.g. physical fitness) than *negative consequences* (e.g. risk of injury) are expected and when the person is self-confident to be physically active on a regular basis (*self-efficacy*). Additionally, the person has to be aware of potential health risks (*risk perception*, such as heart disease). Once an intention is formed, the behaviour has to be realized, which is called volitional phase. Volitional competencies according to the HAPA are action planning, coping planning, and action control. Within an *action plan*, a person has to specify, when, where, and how he or she wants to implement the behaviour (e.g. ‘I plan to go cycling, every Tuesday at 6 pm’). As plans can always be challenged by situational barriers (e.g. rainy weather, one’s own bad mood or the cosy couch at home [25]), the person must have effective *coping plans* in order to overcome these barriers and stick to the exercise intention (e.g. social commitment, environment control, see [25]). The post-intentional phase should be accompanied by consecutive action control [26], through which ‘the ongoing behaviour is continuously evaluated with regard to a behavioural standard’ ([24], p.165). Lastly, there are two kinds of self-efficacy that are important for intention implementation: maintenance and recovery self-efficacy [24]. By explicating post-intentional processes, the HAPA extends the scope of other behaviour change models (e.g. [23, 27, 28]). Overall, the HAPA provides a flexible, theoretical framework that can be used to describe, explain and predict changes in health behaviours. It has already been used successfully in numerous health promotion studies, for example to promote physical activity [26], healthy eating [29] or flu vaccine uptake [30]. Yet, no HAPA-based physical activity intervention in students has been evaluated.

Based on the HAPA, we developed a web-based psychological training called ‘InterAKTIV Sport’ (German title). The training consists of several web-based self-help modules targeting at motivational and volitional competences (cf. methods section). It aims at promoting exercise behaviour as a subset of physical activity. Physical activity is defined as any (physical) activity that requires energy and is caused by the skeletal muscles [10]. Exercise, on the other hand, covers only those physical activities that are structured, planned and repetitive with the goal of improving or maintaining physical fitness [10]. The intervention focuses concisely on exercise, as exercise is supposed to be more tangible for young adults than “physical activity” in general. Many students are familiar with exercise groups and clubs from their adolescence, but often they lost their relatedness when transiting into student life [31].

The study aims at investigating the effects of the web-based HAPA intervention on physical activity in students (primary outcome). We expect that participants of the intervention group will be more physically active than a waitlist control group at post-intervention and 3 months follow-up. Additionally, we investigate the effects of the intervention on motivational and volitional variables proposed by the HAPA model (secondary outcomes). Quality of life and depressive symptoms are investigated with an explorative approach.

## Methods

### Design

A two-armed randomized controlled trial (RCT) of parallel design is conducted in order to investigate the research questions. Participants are recruited via the internet platform StudiCare (www.studicare.com). All students registering for the study and giving informed consent have access to the baseline assessment (t1). Individuals fulfilling inclusion criteria (student status, older than 18 years, exercising less than three times a week) are randomized to either the intervention group or the control group. There are no further exclusion criteria in order to enable as many students as possible to engage in the training and to achieve a high external validity. Participants in the intervention condition will immediately receive access data for the web-based intervention, while participants in the control group do not receive the intervention until follow-up assessment (t3). For the control group access to the web-based intervention is activated after completing the follow-up assessment (t3). After 4 weeks, post-assessment (t2) is performed in both groups followed by a follow-up assessment (t3) 3 months later.

The RCT is conducted in accordance with the CONSORT 2010 Statement [32] and the extension of the CONSORT Statement for pragmatic effectiveness studies [33]. The documentation of the intervention follows the guidelines for implementation and documentation of web-based intervention studies [34]. The study has been approved by the data protection office and the ethics committee of the University of Freiburg (No.: 504/18) and the trial is registered in the German clinical trials register under DRKS00016889. With respect to trial auditing, the Project Management Group (LKr, JB) meets weekly over the course of the trial to oversee conduct and progress of recruitment. An independent data monitoring committee is not implemented, as “InterAKTIV Sport” is a low-risk intervention. Deviations from the study protocol will be fully documented and disclosed in further publications. In case of deviations, the protocol in the clinical trial registry will be updated.

### Recruitment

Participants are recruited via the StudiCare homepage (https://www.studicare.com). StudiCare.com is part of an international research and collaboration project that aims at improving students’ health through web-based interventions [35]. Various health-oriented web-based trainings are presented on the StudiCare homepage. All trainings are free-to-use for students who agree to participate in evaluation studies. The StudiCare project comprises a steadily growing network of almost 20 cooperating universities in Germany, Austria, and Switzerland. The universities regularly inform their students via various channels (e.g. e-mail newsletters, press releases, social media posts) about the possibility of participating in online training courses as part of scientific studies. Some universities also refer to the StudiCare offers on their websites. The university cooperation partners are usually the SGM or psychosocial counselling centres, which also recommend the StudiCare training courses directly to students seeking advice. Thereby, over 400,000 students regularly receive information about new studies on StudiCare. For each study, students can access an online registration form. Once the students’ contact details have been received for our study, the study team will send them detailed study information and a link to an informed consent form. The link leads to the homepage of our study where students give their informed consent online before entering the trial. Recruitment started on March 13, 2019, and is expected to end in October 2020. The expected ending date is only approximative, as the pace of recruitment is dependent of the advertising activities at the partner universities of StudiCare and cannot be influenced by the study team.

### Randomization and blinding

An independent researcher of the Methodological Support Centre of the Rehabilitation Research Network Freiburg, who is not elsewhere involved in the study, prepared randomization and allocation of participants in advance. As a means of randomization, an automated computer-based system is implemented (https://www.sealedenvelope.com/) using permuted block randomization with variable block sizes of 4, 6, and 8 (randomly arranged), in a ratio of 1:1. Participants are informed about their assignment to the intervention or control condition. The means for blinding in this study are limited. Still, data analysts will be blinded by creating syntaxes before adding the treatment condition variable to the data set. Any contact between the research team and study participants (e.g. online assessments, e-mail reminders) is standardized for all study participants (IG and CG) and reduced to a minimum.

#### Intervention condition

Following randomization, participants in the intervention group receive access to the web-based training ‘InterAKTIV Sport’. Participants’ access to other exercise programmes (e.g. university sports programme, personal training) is not restricted. ‘InterAKTIV Sport’ consists of an introductory module, four main modules (1: motivation and objectives, 2: concrete planning, 3: barriers management, and 4: action control), and a final module (see Table 1). The components of the training are derived from the HAPA model.

**Table 1:** Module of the web-based intervention ‘InterAKTIV Sport’

Module	Topic	Content
Intro	Introduction	- Information about the course of the training - Technical information
1	Motivation and objectives	- Formulating an exercise goal - Reflecting positive and negative consequences of the exercise goals - Intention formation
2	Action planning	- Concrete planning
3	Barrier management	- Identification of situative barriers - Development of strategies to cope with barriers
4	Action control	- Self-monitoring, self-evaluation, self-reinforcement - Coping with lapses into inactivity
Final	Completion	- Review of the intervention

Each module closes with a homework assignment encouraging the implementation of the new skills in daily life. New modules are available on a weekly basis, given that the participant has completed the previous chapter. The introduction and final module can be completed on the same day as the first or last content module. The training lasts for 3 weeks if a participant completes all modules within the recommended timeframe. To enhance adherence [36], participants are sent e-mail reminders after 3, 7, and 10 days when activated modules are not completed. In the last reminder, they are informed that their account will be deactivated within 4 days if they will not login again. Participants’ access to the intervention platform closes with sending the follow-up assessment.

The training was generated with the e-learning software Articulate Storyline 360. It is available on our own server with a pre-installed ILIAS application [37] which we use for enrolment and management of participants. Login on the platform takes place via personal access data and is open all the time. The usability of the intervention was tested in a pilot study [38]. Participants’ feedback resulted in an improved version of the intervention.

#### Control condition

**Fig. 1:**
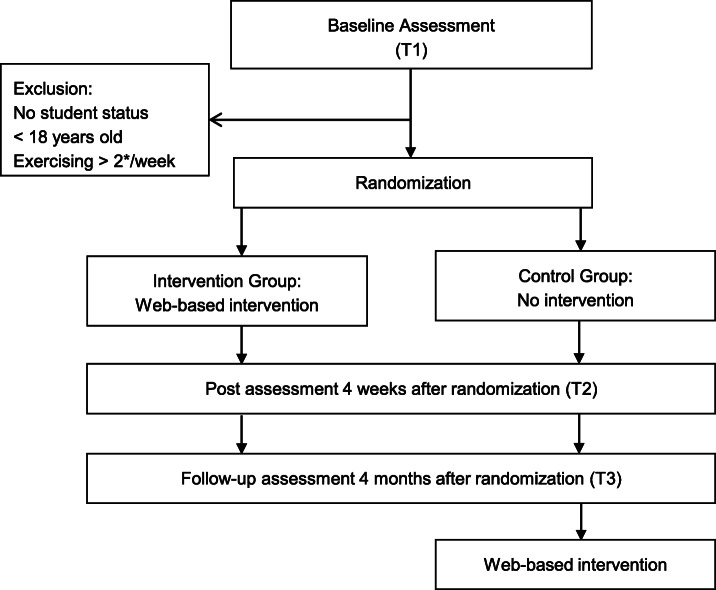
Study flow

The control group receives the intervention after completing follow-up assessment. Control group participants are told that they will get access to the intervention after the follow-up assessment. No further instruction is given what to do—or not to do—during this time. Participants’ access to other exercise programmes (e.g. university sports programme, personal training) is not restricted. This is equivalent to “treatment as usual” (TAU) in medical efficacy studies. A detailed description of TAU is assessed at post-treatment. Figure 1 shows the study flow.

### Proposed sample size

The sample size calculation is based on the difference in change in the primary outcome (physical activity) from pre- to post-assessment in both treatment arms (intention-to-treat analyses). Based on previous studies on web-based physical activity interventions in students, mean effect sizes of around d = 0.5 are expected in per-protocol analyses [18, 22, 39]. In order to detect such intervention effects in two-sided significance testing (α = .05) with a power of 80%, a sample size of 128 participants is required. Taking into account an expected study drop-out of about 20%, 160 participants must be included in the study. This sample constitutes the ITT sample. Adjustment for baseline physical activity scores will further improve the power of the primary comparison.

### Outcome measures

All assessments take place on the online platform. The primary outcome of the effectiveness analyses is physical activity as broader construct which ensures comparability with other studies [40]. Secondary outcomes are outcomes more closely related to the intervention (exercise, motivational and volitional determinants of exercise) as well as quality of life and depressive symptoms (see Table 2 for an overview).

**Table 2:** SPIRIT figure and overview of measures

TIMEPOINT	Measures	Enrolment (T0)	Baseline (T1)	Allocation	Post-Treatment (T2)	Follow-Up (T3)
ENROLMENT:						
Informed consent		x				
Eligibility screen			x			
Randomization				x		
INTERVENTIONS:						
Intervention group (web-based intervention)						
Control group						
ASSESSMENTS:						
Physical activity	IPAQ-SF [41]		x		x	x
Amount of exercise	BSA [42]		x		x	x
Intention strength	[43]		x		x	x
Outcome expectations	[43, 44]		x		x	x
Self-efficacy (motivational, maintenance, recovery)	[45]		x		x	x
Risk perception	[24]		x		x	x
Action planning	[43]		x		x	x
Coping planning	[25]		x		x	x
Situational barriers	[25]		x		x	x
Social support	[46]		x		x	x
Depressive symptoms	CES-D [47]		x		x	x
Quality of life	WHOQOL-BREF [48]		x		x	x
Sociodemographics	[49]		x			
Stage of change	[50]		x			
Self-control competence	VCQ (subscales planning and spontaneity [51])		x			

#### Primary outcome

Physical activity is assessed using the International Physical Activity Questionnaire – Short Form (IPAQ-SF, German short version; [41]). Seven items measure on how many days and in what extent vigorous activity, moderate activity and walking activity were performed during the last 7 days. For generating the total physical activity score (Met-minutes/week), the IPAQ protocol is used. The validity of the IPAQ is good and its retest reliability is .80 [52].

#### Secondary outcomes

*Amount of exercise* is measured with the Physical Activity, Exercise, and Sport Questionnaire (German acronym BSA “Bewegungs- und Sportaktivitätsfragebogen” [42]). Participants indicate if they performed one or more exercise activities within the last 4 weeks. They can specify up to three different activities as well as the frequency (monthly) and the duration (of each episode) of the activity. The calculated BSA score reflects the weekly amount of exercise.

*Intention strength* is assessed using the item “How strong is your intention to exercise regularly during the next 4 weeks” [43]. Response format is on a six-point scale ranging from 0 (“not at all”) to 5 (“very strong”).

*Positive and negative outcome expectations* regarding exercise are assessed using existing scales [43, 44] adjusted to our sample of young adults. More precisely, we excluded all items targeting specifically orthopaedic patients. Ten negative outcome expectations (e.g. “If I exercise regularly, I could hurt myself”) and nine positive outcome expectations (e.g. “If I exercise regularly, I become more confident”) are cumulated into one score. Response format of the items is on a four-point scale ranging from 1 (“I do not agree”) to 4 (“I agree”).

*Motivational self-efficacy* is measured using the item “I am confident that I will be exercising regularly in the next 4 weeks” [24]. Response format is on a four-point scale (1=”I don’t agree” to 4=”I agree). Accordingly, the two facets of *volitional self-efficacy* are measured using the same item format. *Maintenance self-efficacy* is assessed with the item: “I am confident that I will exercise regularly even if it takes some time until it becomes a routine” [24]. *Recovery self-efficacy* is assessed with the item: “I am confident that I am able to resume my regular exercise, even if I have relapsed several times” [24].

*Risk perception* is measured with three items that are cumulated into a mean score [24]. Each item assesses the agreement to the following statement: “The likelihood that I develop a [cardiovascular disease / orthopaedic diseases / metabolic disease] is high”. Response format is on a four-point scale ranging from 1 (“I do not agree”) to 4 (“I agree”).

*Action planning* is assessed by asking the participants if they already have a detailed plan on when, where, how, with whom and how often they will execute their exercise activities [43]. A sum score ranging from 0 to 10 is calculated by adding up the positive answers for two activities.

*Situational barriers* are assessed with the barrier scale of Krämer and Fuchs [25]. Based on the mean score of 17 items, it is measured how many internal barriers (e.g. “I was tired”) and external barriers (e.g. “the weather was bad”) the participant experienced within the last 4 weeks (1 = “not at all” to 4 = “very much”). All items were introduced by the phrase: ‘How strongly did the following situations keep you from exercising within the last 4 weeks?’ Responses were given on a four-point scale ranging from 1 (‘not at all’) to 4 (‘very much’).

*Coping planning* is assessed with the barrier management scale of Krämer and Fuchs [25]. 15 items are used to assess the coping strategies that participants use in order to exercise despite situational barriers. The launching phrase (“In order to stick to your scheduled exercise program…”) is followed by 15 possibilities to deal with barriers (e.g. “…, I remind myself of the benefits of exercising”). Participants report for each item to what degree they agree with the statement (1=”I do not agree” to 4 = “I agree”). A mean score of the items is calculated.

*Action control* covers three facets [24]: self-monitoring is assessed with the item “I have regularly monitored whether I exercised enough”. Awareness of standards is assessed with the item: “I have often thought of my exercise intention”. Self-regulatory effort is assessed with the item “I have taken care to exercise as much as I had intended”. The response format is on a four-point scale ranging from 1 (“I don’t agree”) to 4 (“I agree”).

*Social support* regarding exercise is assessed using the scale developed by Fuchs [46]. The launching phrase (“People in my social environment…”) is followed by seven different items (e.g. “… exercise with me”). Response format is on a four-point scale ranging from “(almost) never” to “(almost) always”.

*Depressive symptoms* are assessed with the Center for Epidemiologic Studies Depression Scale (CES-D scale, German version by [47]). The CES-D scale consists of 20 depressive symptoms that are rated on a four-point scale (0=“rarely or not at all”; 1=“sometimes”; 2=“often”; 3=“most of the time”).

*Quality of life* is assessed with the short version of the World Health Organization Quality of Life Scale (WHOQOL-BREF [48]). It consists of 26 items that are covering four different quality of life domains. In this study, we only investigate three domains: physical health (e.g. “Do you have enough energy for everyday life?”), psychological health (e.g. “How much do you enjoy life?”) and social relationships (e.g. “How satisfied are you with your personal relationships”). The domain “environment” is not relevant for our sample and therefore is not assessed in favour of conciseness of the questionnaire. The 26 items are rated on a five-point scale for the last 2 weeks [48]. Three domain scores will be calculated.

#### Sample characteristics

*Sociodemographic characteristics* (e.g. age, gender, height, weight, marital status) are assessed according to the recommendations of Deck and Röckelein [49]. *Stage of change* is assessed with a single item [50]. *Self-regulatory competencies* is covered using the subscales planning and spontaneity of the Volitional Components Questionnaire (VCQ [51]).

### Statistical analyses

According to CONSORT statement [32], sample characteristics of both subgroups will be contrasted descriptively in order to detect relevant differences between the two samples. All analyses for the primary and secondary outcomes will be performed with an alpha level of 5% which means that the analyses for secondary analyses will be explorative. Outcome analyses will follow an intention-to-treat principle by including all randomized participants into the analyses. Outcomes will be analysed using a linear mixed model handling missing data under the assumption that data are missing at random. All participants and data points will be included in the mixed model analyses. The mixed model for the primary outcome (physical activity) will include group, time, and the interaction of group and time as fixed effects and participant and university as random effect. Analyses will use restricted maximum likelihood estimation (REML). For repeated measures (time), an autoregressive covariance structure with heterogeneous variances is assumed. Secondary outcomes will be analysed accordingly. We will calculate between-group effect sizes for the outcomes using the post-intervention estimated means and their pooled observed standard deviation. Additional per protocol analyses will include only those participants completing all three assessments (IG and CG) and at least three intervention modules (IG). For the main outcome, potential moderators will be analysed exploratively by including the following variables and all possible interactions in the mixed model: gender, body mass index, depressive symptoms, self-control competence and recruiting season (separate mixed model for each moderator). All analyses will be conducted using IBM SPSS.

## Discussion

Web-based interventions are a low-threshold, cost-effective way to disseminate physical activity among students. This study is the first high quality RCT to investigate the effect of a web-based physical activity intervention on amount of physical activity among students in Western countries. The implemented web-based intervention is based on the HAPA model of health behaviour change and targets psychological determinants of physical activity such as self-efficacy or action planning. We expect the intervention group to benefit from the web-based intervention with regard to physical activity, at post intervention and 3 months follow-up. In an exploratory approach, we also analyse motivational and volitional determinants of exercise, quality of life and depressive symptoms. The results of this study will be of great relevance for student health campaigns, as they reflect the effectiveness of a self-help intervention for young adults seeking support in establishing a healthy lifestyle.

From a lifespan perspective, it is important to help students find their way into regular physical activity [53]. A special characteristic of the intervention is the focus on exercise behaviour. For our participants, the intervention does not explicitly target health or health behaviour, but rather frames exercise as an activity full of pleasure, vividness and social experience. By activating motives that are relevant for younger adults, the intervention aims at activating the participants without expatiating a health perspective. As Kazdin and Blase [54] point out, innovative approaches are needed to decrease the global burden of diseases.

This study is a high quality RCT with three assessment points and intention-to-treat analysis meeting the state-of-the-art of randomized controlled studies [32]. Recruitment covers almost 20 universities in three countries, leading to high external validity of the results. The intervention is theory-based and was improved after a pilot study. Yet, the study has limitations that deserve note. The inclusion criterion concerning exercise at baseline is set at 0–2 times of exercise per week. It may be that the intervention effectiveness varies between no- und low-exercisers. We use validated scales and items that have been used in former studies. However, validation studies did not particularly focus on young adults. All outcome measures were based on self-reports only. Self-reports underlie potential biases such as social desirability or memory effects. Objective measures of physical activity were not implemented considering the organizational effort and the resources available. A replication study using objective measures would be beneficial. As the study is too underpowered to properly analyse potential moderating variables, the moderator analyses are explorative.

With regard to the intervention, we do not provide any guidance in terms of content for the intervention participants [55]. This may restrict the range of effectiveness of our physical activity interventions. Last, the recruitment of participants has started before submitting the study protocol. However, the study has been registered before the beginning of recruitment and no changes have been made to the registry.

### Trial status

Protocol version number: 1 (November 18, 2019)

First day of recruitment: March 13, 2019

Expected end of recruitment: October 30, 2020

## Data Availability

Not applicable

## Abbreviations

BSA: Bewegungs- und Sportaktivitätsfragebogen (German acronym for Physical Activity, Exercise, and Sport Questionnaire)
CES-D: Center for Epidemiologic Studies Depression Scale
CG: Control group
HAPA: Health Action Process Approach
IG: Intervention group
RCT: Randomized-controlled trial
VCQ: Volitional Components Questionnaire
WHOQOL-BREF: World Health Organization Quality of Life Scale, short version
